# Parent-child discrepancies in reports of pre- and early adolescent level of personality functioning

**DOI:** 10.3389/fpsyt.2026.1773598

**Published:** 2026-03-05

**Authors:** Kiran Boone, Jessica LaRocca, Kennedy M. Balzen, Carla Sharp, Dara E. Babinski

**Affiliations:** 1 Department of Psychology, University of Houston, Houston, TX, United States; 2 Department of Psychiatry and Behavioral Health, Penn State College of Medicine, Hershey, PA, United States

**Keywords:** alternative model for personality disorders, early adolescence, informant discrepancy, level of personality functioning, personality disorder, pre-adolescence

## Abstract

Given the research consensus that personality disorder often onsets in adolescence, more work is needed to investigate parent-child discrepancies in reporting on personality disorder, particularly during the pre- and early adolescent period when more significant impairment in personality functioning may be developing or can already be observed. The current study examined concordance of parent- and child-reported level of personality functioning (LPF, as defined in the DSM-5 Alternative Model of Personality Disorders) among pre- and early adolescents and examined the extent to which this concordance was associated with clinically relevant outcomes. Participants included *N* = 432 children between the ages of 10 and 15 years from three samples oversampled for psychopathology symptoms and their parents. Children and their parents reported on child impairment in personality functioning with the Level of Personality Functioning Scale Brief Form 2.0. Outcomes included parent-reported caregiver strain, parent-reported child functional impairment, and child-reported history of thoughts and behaviors related to suicide and non-suicidal self-injury. Latent profile analysis was conducted to identify profiles of children based on patterns of convergence and divergence between parent- and child-reported LPF. Profile membership was then used to predict outcomes. A four-profile model, with two parent-child convergent and two parent-child divergent profiles, demonstrated the best fit. Convergence on high impairment in LPF demonstrated the strongest associations with outcomes. Divergent profiles also demonstrated stronger associations with outcomes reported by the informant who had endorsed higher impairment in LPF. Findings suggested that both parent- and child-reported LPF, and the degree of their concordance, may have unique value for predicting clinically important outcomes in pre- and early adolescence. Research and clinical practice utilizing new dimensional approaches with adolescents may therefore benefit from multi-informant assessment of personality functioning.

## Introduction

Personality disorder is a serious mental health disorder associated with elevated psychosocial and functional impairment, multiple psychiatric comorbidities, high rates of suicide attempt and non-suicidal self-injury (NSSI), and high cost to the health care system (1–11). These outcomes reflect a significant public health issue necessitating greater attention to the prevention and early intervention of personality disorder. A now extensive evidence base supports the validity of personality disorder diagnosis in adolescence and that personality disorder in fact typically onsets during adolescence (12–17). Pre- and early adolescence is therefore a critical developmental period for early intervention, during which personality functioning may be more malleable and treatment may be particularly helpful for supporting young people in healthy personality development. A better understanding of personality pathology in pre- and early adolescence is crucial for identifying appropriate early-life treatment targets. While a few self-report measures have been developed and validated for the assessment of personality pathology during late childhood, pre-adolescence, and early adolescence (18–20), obtaining reports from multiple informants could offer additional, valuable insights. However, in order for these reports to be clinically useful, clinicians must have a guiding framework for interpreting multiple informant reports of personality pathology, particularly when these reports are discrepant (21).

It has been well established that children and their caregivers, as well as other informants such as teachers and peers, often provide discrepant reports of child behavior and psychopathology (22, 23). Collecting information from multiple informants is standard clinical practice in the psychiatric assessment of children and adolescents, yet guidelines for addressing informant discrepancy remain unclear and providers are often left unsure of how to appropriately interpret multi-informant reports. Traditionally, the principle of converging operations has been utilized to address data discrepancies (24). Related to the concept of convergent validity, converging operations asserts that the validity of a construct and its operationalization is dependent on the degree to which distinct channels of information converge and draw similar conclusions (24, 25). While often discussed with regards to different measures of the same behavior (i.e. convergence between two self-reports or between a self-report and an interview), this expectation of convergence has been extended to multiple informant reports, such that if multiple informants are evaluating the behavior or symptoms of the same individual, then each of the reports should align with one another. In cases of discrepancy, clinicians may discount reports from the informant judged to be less plausible, less reliable, or less insightful in order to increase assessment validity (26, 27). In clinical evaluations of child and adolescent mental health, children are often viewed as the less reliable or credible source when caregivers report more concern about emotional or behavioral difficulties (26), when family functioning is better (28), or caregivers are more educated, although differences in perceived credibility may vary across disorders and lessen as children age into adolescence (27). Notably, while poor reliability does interfere with validity in assessment, research indicates that informants expected to be less credible or reliable by clinicians actually tend to demonstrate similar or higher internal consistency in their reports (27, 29). Further, ample research suggests that discrepancy in reports of child behavior and psychopathology tends to be the rule, not the exception, such that rates of correspondence remain low to moderate at best (22, 23, 30). The widespread nature of informant discrepancies has challenged the utility of relying solely on the principle of converging operations, calling for an updated theoretical and methodological framework (24).

As such, in our discussion of multi-informant discrepancy, we draw on the operations triad model proposed by De Los Reyes and colleagues (31), which expands upon and addresses the conceptual shortcomings of converging operations. The operations triad model establishes two alternative principles for the interpretation of informant discrepancies. First, the principle of diverging operations highlights conditions in which report discrepancies reflect meaningful variation in the construct being assessed, resulting from differences in the context or setting in which a behavior, symptom, or other manifestation of the construct is observed, or differences in the level of insight that an informant would be expected to have into the behavior or symptom. Even when the same behavior is observed by multiple informants, these informants may also differ in their interpretation of the behavior, and these different interpretations could provide unique and valuable information. The second principle is compensating operations, which highlights conditions in which report discrepancies do not reflect meaningful variation and are instead based on differences in measurement validity or reliability between informant reports, methodological differences between informant reports that might contribute to effects of shared methods variance, or differences in informant characteristics that are not relevant to the construct of interest. Thus, the operations triad model calls upon researchers and clinicians to recognize meaningful sources of informant discrepancy and to differentiate these sources from contributions of measurement error.

With the operations triad model providing a conceptual framework for interpreting informant discrepancy, a growing literature has examined informant discrepancies between parents’, teachers’, children’s, and peers’ reports – in addition to laboratory-based measures and interviewer ratings – on a wide range of internalizing (29, 32–34, 36, 35–37) and externalizing (29, 35, 37–40) disorders and symptoms, as well as autism spectrum disorder (41) and pediatric quality of life (42). Findings have suggested that informant discrepancies often reflect meaningful differences in the contexts in which behaviors and symptoms are observed and have potentially important implications in the assessment and treatment of psychopathology, aligning with the principle of diverging operations (23, 24, 31, 43, 44). Collectively, this body of work demonstrates how patterns of informant discrepancy can provide useful knowledge in the study of early adolescent psychopathology.

However, the application of these approaches to the investigation of personality pathology in youth remains limited, and extant work has focused mostly on discrepancies in borderline personality disorder (BPD) features. In line with findings on other psychopathology, this research has found only modest to moderate convergence between child-reported and parent-reported BPD features in adolescence, and that relative convergence versus divergence in child- and parent-reported BPD features is predictive of clinically relevant outcomes (45–48). Specifically, Wall and colleagues (46) found that convergent parent-child reports of high BPD features were associated with greater psychiatric severity compared to divergent reports where parents reported more BPD features than children. Another study by Jørgensen and colleagues (47) indicated that greater discrepancies between caregiver and adolescent reports of adolescent BPD features, where adolescents reported more BPD features than parents reported, were significantly associated with more adolescent-reported attachment problems in both peer and caregiver-adolescent relationships. Additionally, Vanwoerden and colleagues (48) suggested that parent-child agreement may be highest when reporting on the externalizing symptoms of BPD. While this sparse literature has demonstrated the potential value of multi-informant assessment of personality pathology in adolescence, more work is needed to further elaborate parent-child discrepancies in personality pathology broadly, particularly as the field is undergoing a shift away from the categorical framework of personality disorders and toward a dimensionally informed model of personality disorder (49). The Alternative Model for Personality Disorders (AMPD) was introduced as a dimensional framework for personality disorder in the fifth edition of the Diagnostic and Statistical Manual of Mental Disorders (DSM-5) (50) in response to the many noted limitations of the categorical model with regards to diagnostic validity and clinical utility (51–53). The entry criterion for personality disorder diagnosis in the AMPD is represented by Level of Personality Functioning (LPF), a dimensional construct characterized by impairments in self- and interpersonal functioning that are common and core to all personality disorders (54, 55). LPF allows for subthreshold impairment to be identified and thus has been argued to be a developmentally sensitive construct, capturing the emergence and onset of personality disorder during adolescence (56–58). Indeed, several interview-based, self-report, and parent-report measures of LPF have been developed for use and validated in adolescence (18, 20, 58–60). Therefore, the assessment of LPF in early adolescence may aid early detection of personality disorder and advance early intervention.

However, research has yet to examine discrepancies between parent- and child-reported LPF and how such informant discrepancies may guide clinical decision making. These discrepancies may be particularly valuable to examine in the pre- and early adolescent period, when more significantly impaired LPF may be developing or can already be observed. Thus, the primary aim of the current study was to examine the patterns of convergence and divergence between parent and pre-/early adolescent reports of LPF and the associations of these patterns with clinically important outcomes that are particularly salient to personality disorder. These include caregiver strain, child functional (i.e. social and academic) impairment, and thoughts and behaviors related to suicide and NSSI because of their potential significance for prognosis and treatment and their known associations with personality dysfunction (9, 11, 49, 61–65). Specifically, prior work has demonstrated greater functional impairment and higher rates of suicide- and NSSI-related thoughts and behaviors among adolescents with personality disorder compared to adolescents with other psychiatric diagnoses (66, 67), as well as higher rates of distress and caregiving burden among carers of individuals with personality disorder or personality disorder features compared to carers of individuals with other mental health difficulties (68, 69). It is therefore worth examining how convergence versus divergence in parent- and child-reported LPF might be associated with these outcomes.

To achieve our goals, first, a person-centered, exploratory latent profile analysis approach was utilized to identify subgroups of children based on patterns of convergence and divergence between parent- and child-reported LPF. Once the best fitting latent profile model was identified and participants were assigned to latent profiles, we examined associations between latent profile membership and potentially clinically relevant outcomes, including parent-reported child functional impairment, parent-reported caregiver strain, and child-reported history of thoughts and behaviors related to suicide and NSSI.

While the latent profile analysis was exploratory, we expected based on prior work on informant discrepancies in psychopathology (e.g. 40, 41, 70) that at least four latent profiles would be identified, with one profile reflecting parent-child convergence on low impairment in LPF, one profile reflecting convergence on high impairment in LPF, one profile reflecting divergence with high impairment in LPF reported by parents and low impairment in LPF reported by children, and one profile reflecting divergence with high impairment in LPF reported by children and low impairment in LPF reported by parents. We did not have any *a priori* hypotheses as to how these profiles would differ based on demographic variables. Regarding associations with outcomes, we hypothesized that membership in profiles characterized by parent-child convergence on high impairment in LPF or by parent-reported higher impairment in LPF would be associated with higher caregiver strain and higher child impairment compared to membership in other profiles. We further hypothesized that membership in profiles characterized by parent-child convergence on high impairment in LPF or by child-reported higher impairment in LPF would be associated with higher reported suicide- and NSSI-related thoughts and behaviors compared to membership in other profiles. Thus, in line with a diverging operations perspective, we expected that both convergent and divergent latent profiles would provide meaningful information about outcomes.

## Methods

### Participants and Procedures

Data were pooled from three studies focused on social functioning and personality development. All studies included parent and youth ratings of LPF as part of their cross-sectional assessment battery as well as additional overlapping demographic and clinical measures. The first study included 87 pre- and early adolescent girls (ages 11–15 years) with and without attention-deficit/hyperactivity disorder (see 71). Data for this study were collected from June 2021 to May 2024. The second study included 110 pre- and early adolescent girls (ages 10–14 years), oversampled for mental health problems to study the development of borderline personality features over one year (see 72). Data for this study were collected from December 2021 to October 2022. The third study included 261 pre- and early adolescent boys and girls (ages 10–14 years), oversampled for mental health problems in a study examining the development of impairment in LPF over two years (see 73). Data for this study were collected from October 2024 to November 2025. For all studies, participants were required to be fluent in English; visual or hearing impairments that may have hindered completing study procedures, IQ < 80 based on the Wechsler Abbreviated Scale of Intelligence – Second Edition (WASI-2), intellectual or developmental disabilities, or lifetime diagnosis of bipolar disorder, autism spectrum disorder, schizophrenia, or other psychotic disorders were exclusionary. A total of 432 participants and caregivers with LPF data available were included in the current analysis. In the current analytical sample, 98.84% of caregivers reported that they were the mother or father of the child participant, while 1.16% reported that they were the grandfather or grandmother of the child participant; the term “parent” is used henceforth to describe caregivers.

Participants completed measures via Research Electronic Data Capture (REDCap), a secure, web-based platform used to administer surveys and manage research data. Questionnaires were completed electronically using a computer, tablet, or smartphone through individualized survey links. Parents and children completed measures separately, and participants were instructed to complete questionnaires privately. Research staff were available to assist with questions as needed. Most participants completed ratings during their in-person study visit, although as needed, participants were allowed to complete assessments at home.

### Measures

#### Level of personality functioning

Children and their parents both completed the Level of Personality Functioning Scale - Brief Form 2.0 (LPFS-BF 2.0), a 12-item measure of self- and interpersonal functioning (74). Items are rated on a 4-point Likert-type scale from 1 (*completely untrue*) to 4 (*completely true*), such that total scores range from 12 to 48. One study providing normative data in adults across the lifespan (75) suggested that total scores of 31 and higher on the LPFS-BF 2.0 may indicate clinically relevant impairment in LPF, and that total scores between 26 and 31 may indicate subclinical, mild impairment in LPF. The clinical cut-off score of 31 was supported in another study of young adults (76), although clinical cutoff data for adolescents is still needed. The LPFS-BF 2.0 has demonstrated strong internal consistency and associations with other measures of personality pathology in adolescents aged 12 to 18 years (20). The original self-report items were modified for parents to report on their child’s LPF. Given the value of computing a sum score for interpretation against suggested clinical cutoff scores and the low rate of missingness in both the child-reported and parent-reported LPFS-BF 2.0 (1.9% of children and 2.8% of caregivers with one missing item, one child with two missing items), missing items were imputed with the mean of that participants’ other items on the LPFS-BF 2.0. A sum score was then calculated for a total impairment score. Internal consistency of the LPFS-BF 2.0 was good for both child report (ɑ = .868) and parent report (ɑ = .875) in the current sample.

#### Parent-reported outcomes

Parents completed a 12-item measure assessing caregiver strain, including objective strain (i.e. disruptions to work or financial strain), subjective internalized strain (i.e. feelings of sadness and worry), and subjective externalized strain (i.e. feelings of anger and resentment) experienced by caregivers of children and adolescents with emotional or behavioral disorders. This measure included the 11 items from the Caregiver Strain Questionnaire - Short Form (CSQ-SF) (77) as well as an additional item (“In general, how much of a toll do your child’s problems take on your family?”) (78). Items are rated on a 5-point Likert-type scale from 1 (*not at all*) to 5 (*very much*). The CSQ-SF has demonstrated invariance across child and caregiver sex and age, excellent internal consistency, and strong associations with parent-reported child psychopathology and impairment (77). The rate of missingness in the current sample was low (2.5% of parents were missing one item). An average across items was calculated for a total caregiver strain score. Therefore, missing items were not imputed with the mean score. Internal consistency of the CSQ-SF was excellent (ɑ = .931) in the current sample.

Parents also completed the Impairment Rating Scale (IRS) (79) to assess child functional impairment. Seven items were drawn from the parent version of the IRS, assessing children’s difficulties with academic progress, self-esteem, relationships with peers, parents, and siblings, overall family functioning, and global problems, and one additional item from the teacher version of the IRS assessing classroom behavior. These eight items were rated on a Likert-type scale from 0 (*no problem*) to 6 (*extreme problem*). However, if the parent reported that the child did not have siblings, the item assessing sibling relationships was left blank. The parent and teacher versions of the IRS have demonstrated strong one-year temporal reliability and strong associations with other measures of child impairment and psychopathology (79). The rate of missingness across items (excluding the item assessing sibling relationships) was low (1.6% of parents were missing one item). An average across items was calculated for a total impairment score. Therefore, missing items were not imputed with the mean score. Internal consistency of the IRS was excellent (ɑ = .931) in the current sample.

#### Child-reported outcomes

Children completed the Self-Injurious Thoughts and Behaviors Interview (SITBI) - Short Form (80, 81), a 72-item structured interview assessing history of thoughts and behavior related to suicide and NSSI. We included children’s answers (no coded as 0, yes coded as 1) to the following questions as individual outcomes (1): Have you ever had thoughts of killing yourself? (suicidal ideation); 2) Have you ever actually made a plan to kill yourself? (suicide plan); 3) Have you ever done something to lead someone to believe that you wanted to kill yourself when you really had no intention of doing so? (suicide gesture); 4) Have you ever made an actual attempt to kill yourself in which you had at least some intent to die? (suicide attempt); 5) Have you ever had thoughts of purposely hurting yourself without wanting to die (for example, cutting or burning)? (thoughts of NSSI); and 6) Have you ever actually engaged in NSSI? (engagement in NSSI). The rate of missingness was low (≤1.6% for each item). Participants who did not provide an answer to any one of these items were excluded from analyses examining that item as an outcome. Therefore, missing items were not imputed.

#### Demographic variables

Parents reported child sex (female coded as 0, male coded as 1), parent sex (female coded as 0, male coded as 1), child age in years, child race and ethnicity, parent educational attainment, parent employment status, and parent marital status.

### Data analysis plan

Pearson’s correlations (including point-biserial correlations as needed) were conducted to examine bivariate relationships between all study variables. A paired-sample *t*-test between child-reported and parent-reported LPF was conducted to determine whether there was a significant difference between their means. Exploratory latent profile analysis (LPA) was conducted using the *tidyLPA* (82) and *mclust* (83) packages in R version 4.4.1. Following guidance by Sinha and colleagues (84) on best practices for LPA, we examined whether assumptions for LPA were met, including normality, homogeneity of variance, and independence of indicator variables. The sample size (*N* = 432) was considered adequate given a suggested minimum sample size of 300 (84). We started by estimating six models, with one up through six latent profiles, and evaluated the optimal number of latent profiles by examining fit indices, model comparisons, and model parsimony. Informed by guidance by Sinha and colleagues (84) and Tein and colleagues (85), we jointly examined the Bayesian information criterion (BIC) and the sample-adjusted BIC (SABIC), where smaller values indicate better model fit to the data, and the bootstrapped likelihood ratio test (BLRT), where significant *p*-values indicate better model fit of a *k*-class model compared to a *k-1* -class model. Akaike’s information criterion (AIC) and entropy were examined but have been suggested to be poorer indicators of fit in LPA (84, 85).

After selecting a model with the optimal number of latent profiles, each participant was assigned to the latent profile to which they had the highest posterior probability of belonging. The mean probability of profile assignment across and within profiles was examined to evaluate the degree of uncertainty in profile assignment. As suggested by Nagin (86), we considered a mean probability of profile assignment of 0.7 or higher as indicating an acceptable degree of uncertainty in profile assignment for proceeding with further analyses. Further, as suggested by Sinha et al. (84), we examined probability of profile assignment at the individual participant level and considered 0.5 or higher as an acceptable cut-off. If any participants’ probability of assignment fell below this cutoff, we reported descriptive statistics and results of primary analyses with and without removing these participants.

After assigning participants to latent profiles, within-profile descriptive statistics were examined. If child sex, parent sex, or child age differed significantly across profiles (as indicated by Pearson’s chi-square test, Fisher’s exact test, or ANOVA respectively), these variables were included as covariates in further analyses predicting outcomes. Other demographic variables were used to describe the sample. We also conducted Pearson’s chi-square test to examine whether study membership (i.e. membership in one of the three studies from which the analytical sample for the current study was drawn) was associated with profile membership, as this could contribute to sampling artefacts rather than true profiles.

Profile membership was then used to predict outcome variables. One-way analysis of variance (ANOVA) was used to examine the associations between profile membership and continuous outcomes or demographic variables, and Tukey’s HSD test was used to probe significant differences *post hoc*. Pearson’s chi-square test (or Fisher’s exact test where cell counts ≤ 5) was used to examine the associations between profile membership and dichotomous outcomes or demographic variables, and pairwise comparisons with Bonferroni correction were used to probe significant differences *post hoc*. If demographic variables significantly differed across latent profiles, we accounted for these variables as covariates via analysis of covariance (ANCOVA) or generalized linear model (GLM) with a binomial distribution as appropriate. Hypotheses and data analyses were preregistered at osf.io/37yzc (87).

## Results

Children included 301 females (69.68%) and 131 males (30.32%). Most children (93.75%) were between the ages of 10 and 13. Children were 86.34% monoracial White, 8.33% multiracial, and 2.55% monoracial Black, and were 8.80% Hispanic. Parents included 395 females (91.44%) and 37 males (8.56%). Parents’ reported educational attainment was as follows: bachelor’s degree (35.50%), master’s degree (23.67%), doctoral degree (10.21%), graduate of two-year college or trade school (12.53%), attended college or trade school but did not graduate (11.14%), high school graduate or GED (6.73%), and did not graduate high school (0.23%). Most parents (95.60%) reported that at least one parent in the family worked full time. Parents’ reported marital status was as follows: married (78.42%), divorced or separated (10.67%), living with a partner (6.03%), single and never married (3.71%), widowed (0.70%), or other (0.46%).

Descriptive statistics for child age and continuous outcome variables are presented in **Table 1**. With regards to suggested cutoff scores on the LPFS-BF 2.0, child-reported LPF was above the subclinical cutoff score for 28.24% of all participants and above the clinical cutoff score for 10.42% of all participants. Parent-reported LPF was above the suggested subclinical cutoff score for 23.38% of all participants and above the clinical cutoff score for 8.10% of all participants. Regarding dichotomous outcome variables, 19.30% of children reported having ever had active suicidal ideation, 4.88% reported having ever made a suicide plan, 5.81% reported having ever made a suicide gesture, 2.33% reported having ever made a suicide attempt, 16.36% reported having ever thought of engaging in NSSI, and 11.06% reported having ever engaged in NSSI.

**Table 1:** Sample descriptive statistics.

Variable	*N*	Minimum	Maximum	*M*	*SD*	Skewness	Kurtosis
Child-reported LPF	432	12.00	42.00	21.231	6.546	0.593	-0.356
Parent-reported LPF	432	12.00	43.00	20.197	6.657	0.718	-0.205
Parent-reported caregiver strain	431	1.00	4.50	1.609	0.684	1.452	1.999
Parent-reported child functional impairment	432	0.00	6.00	1.422	1.387	0.814	-0.141
Child age	432	10	15	11.720	1.228	0.113	-1.040

LPF, level of personality functioning. Higher values on LPF reflect greater impairment in personality functioning.

### Bivariate correlations and differences in mean level of personality functioning

Bivariate correlations indicated statistically significant positive relationships between child-reported LPF and all outcome variables and between parent-reported LPF and all outcome variables except for suicide gesture (**Table 2**). Parent-reported outcomes (caregiver strain and child impairment) were more strongly correlated with parent-reported LPF than child-reported LPF, and child-reported outcomes (suicide- and NSSI-related thoughts and behaviors) were either somewhat more strongly correlated with child-reported LPF or similarly correlated with both child-reported and parent-reported LPF. Older children reported greater impairment in LPF compared to younger children and were more likely to endorse having ever had suicidal ideation, a suicide plan, a suicide attempt, thoughts of NSSI, and having engaged in NSSI. Female children reported greater impairment in LPF compared to male children, but female children also tended to be older than male children in the sample, suggesting a potential confound with age. Female parents reported greater impairment in LPF, caregiver strain, and child impairment compared to male parents. Child-reported LPF and parent-reported LPF were modestly correlated (*r* = 0.267, *p* <.001). The two-tailed paired-sample t-test between parent- and child-reported LPF indicated that means were significantly different (*t* = 2.688, *df* = 431, *p* = .007), though the magnitude of the difference was small (Cohen’s *d* = 0.129, 95% confidence interval [0.035, 0.224]).

**Table 2:** Bivariate correlations.

Variable	1	2	3	4	5	6	7	8	9	10	11	12
1. Child- reported LPF	**-**											
2. Parent- reported LPF	**.267**	–										
3. Caregiver strain	**.230**	**.701**	–									
4. Child impairment	**.254**	**.724**	**.817**	–								
5. Suicidal ideation	**.337**	**.256**	**.183**	**.184**	–							
6. Suicide plan	**.194**	**.239**	**.171**	**.175**	**.463**	–						
7. Suicide gesture	**.154**	.021	.051	.029	**.130**	.036	–					
8. Suicide attempt	**.136**	**.154**	**.107**	**.112**	**.318**	.**625**	.093	–				
9. Thoughts of NSSI	**.311**	**.173**	**.128**	**.111**	**.566**	**.338**	**.213**	**.269**	–			
10. Engaged in NSSI	**.274**	**.118**	**.107**	**.121**	**.398**	**.300**	**.199**	**.242**	**.632**	–		
11. Child age	**.174**	.083	.015	.008	**.127**	**.167**	-.008	**.124**	**.096**	**.134**	–	
12. Child sex	**-.127**	-.044	-.018	.072	-.053	-.008	-.055	.033	-.072	-.037	**-.136**	–
13. Parent sex	.006	**-.114**	**-.141**	**-.144**	-.066	-.031	-.076	.008	-.043	-.053	.057	-.040

Bold type indicates statistically significant correlations (*p* <.05). Child and parent sex were coded 0 (female) and 1 (male). LPF, level of personality functioning; NSSI, non-suicidal self-injury.

### Exploratory latent profile analysis

Regarding assumptions for LPA, both child-reported and parent-reported LPF were approximately normally distributed and not highly correlated, and Levene’s test for homogeneity of variances indicated that their variances were not significantly different (*p* = .591). We then conducted LPA and evaluated fit indices, comparative fit between models, and parsimony across models, starting with six models with one through six profiles each (**Table 3**). The four-profile model had the lowest BIC value. BLRT suggested that the four-profile model provided significantly better fit than the three-profile model, while the five-profile model did not provide significantly better fit than the four-profile model. The six-profile model had a somewhat lower SABIC value than the four-profile model, and also fit significantly better than the five-profile model using the BLRT. Visual examination and examination of the estimated means and variances of the four-profile and six-profile models indicated better separation of profiles in the four-profile model and that the six-profile model contained two highly overlapping profiles. Therefore, to maximize both model fit and parsimony, the four-profile model was selected for further analyses.

**Table 3:** Model fit indices for determining the optimal number of latent profiles.

Number of profiles	AIC	BIC	SABIC	Entropy	BLRT value	BLRT *p*
1	5719.128	5735.402	5722.708	1		
2	5627.051	5655.530	5633.316	0.781	98.077	.010
3	5634.740	5675.425	5643.690	0.716	-1.690	1
4	5585.727	5638.617	5597.362	0.766	55.013	.010
5	5591.684	5656.779	5606.004	0.584	0.0429	.475
6	5575.598	5652.898	5592.603	0.621	22.086	.010

AIC, Akaike’s information criterion; BIC, Bayesian information criterion; SABIC, sample-adjusted Bayesian information criterion; BLRT, bootstrapped likelihood ratio test.

Each participant was assigned to the latent profile to which they had the highest posterior probability of belonging. The mean assignment probability across the four profiles was 0.879, and the mean probability of assignment for each profile was 0.818 (SD = 0.190), 0.919 (SD = 0.120), 0.855 (SD = 0.144), and 0.839 (SD = 0.152) for profiles 1 through 4, respectively. Given Nagin’s (86) suggested cutoff of 0.7 or higher for acceptable mean assignment probability, the level of ambiguity in profile assignment was considered acceptable. However, there were twelve participants for whom the assignment probability was lower than 0.5, indicating considerable uncertainty in profile assignment for these participants. We therefore calculated descriptive statistics and conducted primary analyses when removing these twelve participants. Effect sizes remained similar, and statistical significance decisions after correction for multiple comparisons remained the same with the exception of two *post-hoc* comparisons. These results are presented in the **Supplementary Material** (**Supplementary Table 1**, **S2**, **S3**).

**Figure 1:**
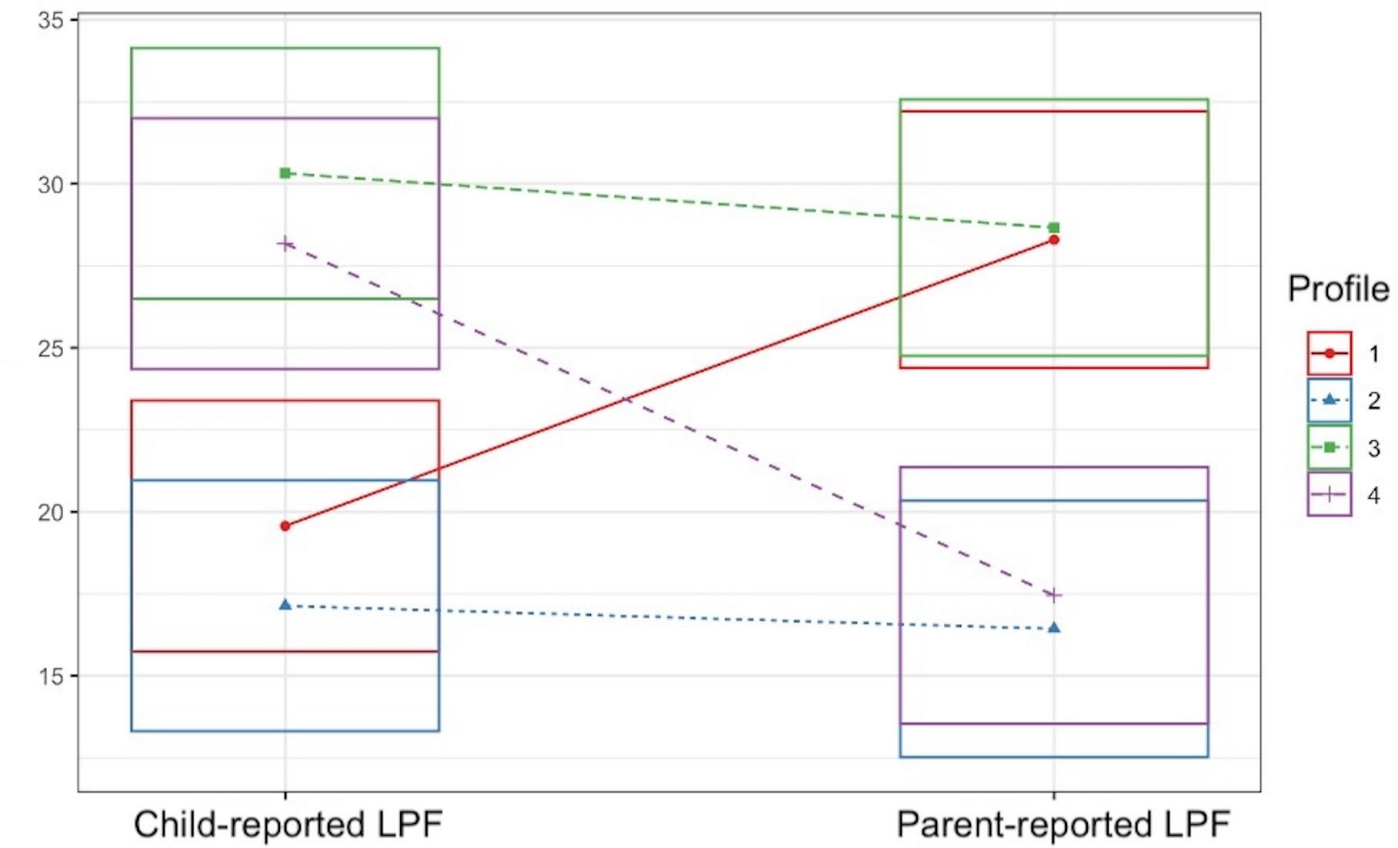
Univariate plot of the four-profile solution emerging from exploratory latent profile analysis. Boxes encompass one standard deviation above and below the mean. LPF, level of personality functioning.

**Figure 2:**
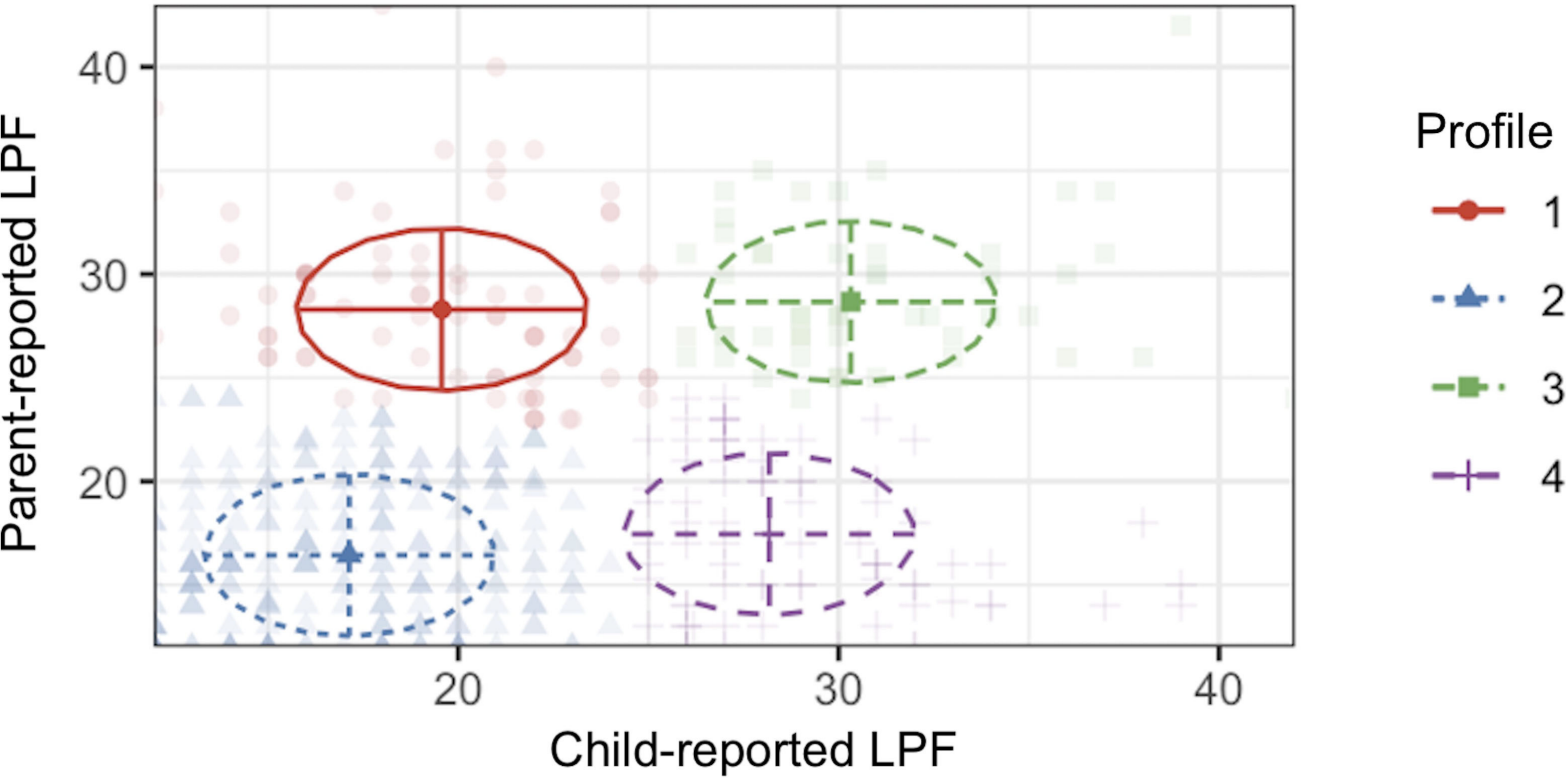
Bivariate plot of the four-profile solution emerging from exploratory latent profile analysis. Lines emanating from center points represent one standard deviation from the mean. Ellipses represent correlation estimates. Raw data is plotted according to assigned profile membership, with higher opacity reflecting higher probability of profile membership. LPF, level of personality functioning.

**Figure 3:**
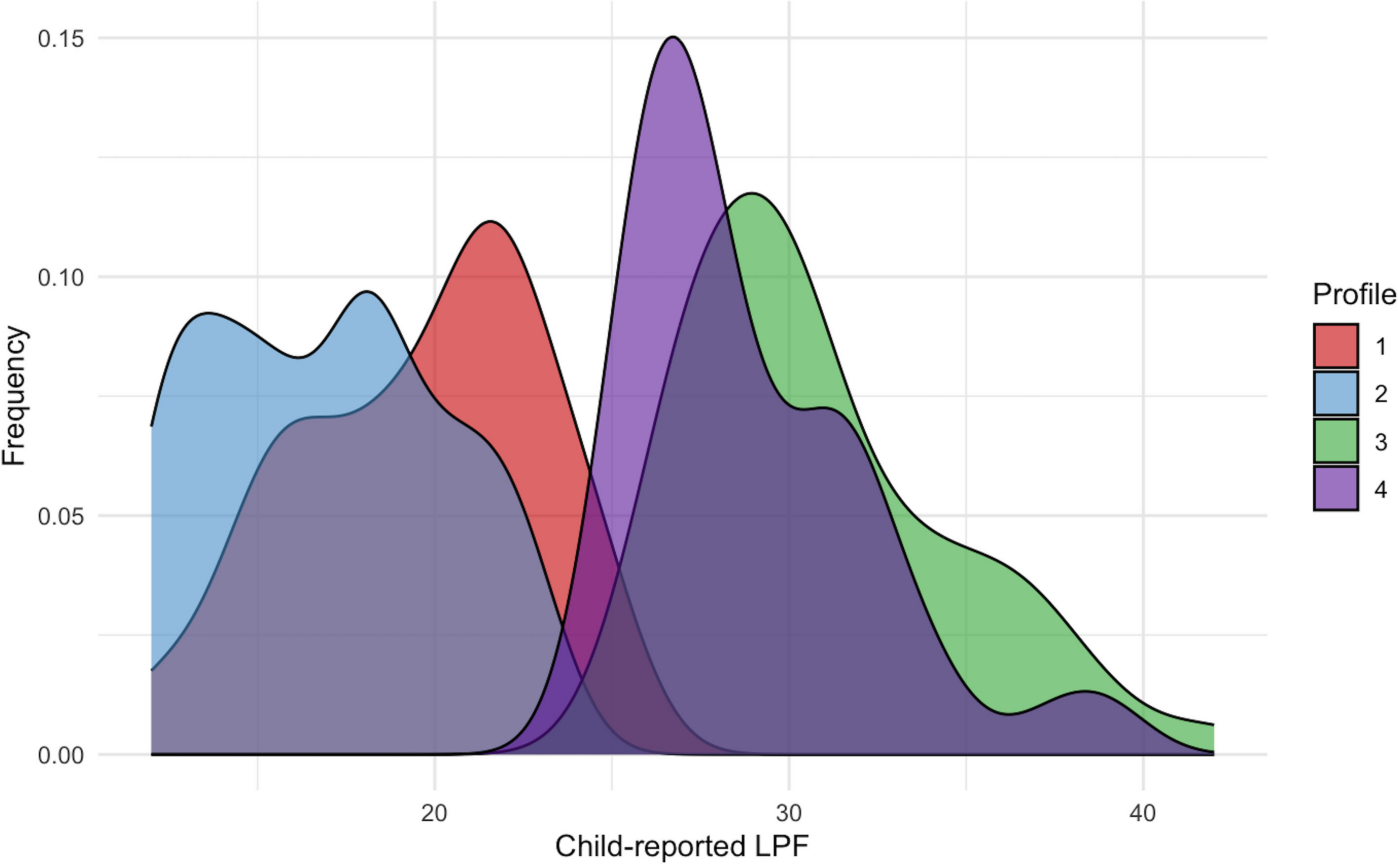
Density plots of child-reported level of personality functioning within latent profiles. LPF, level of personality functioning.

**Figure 4:**
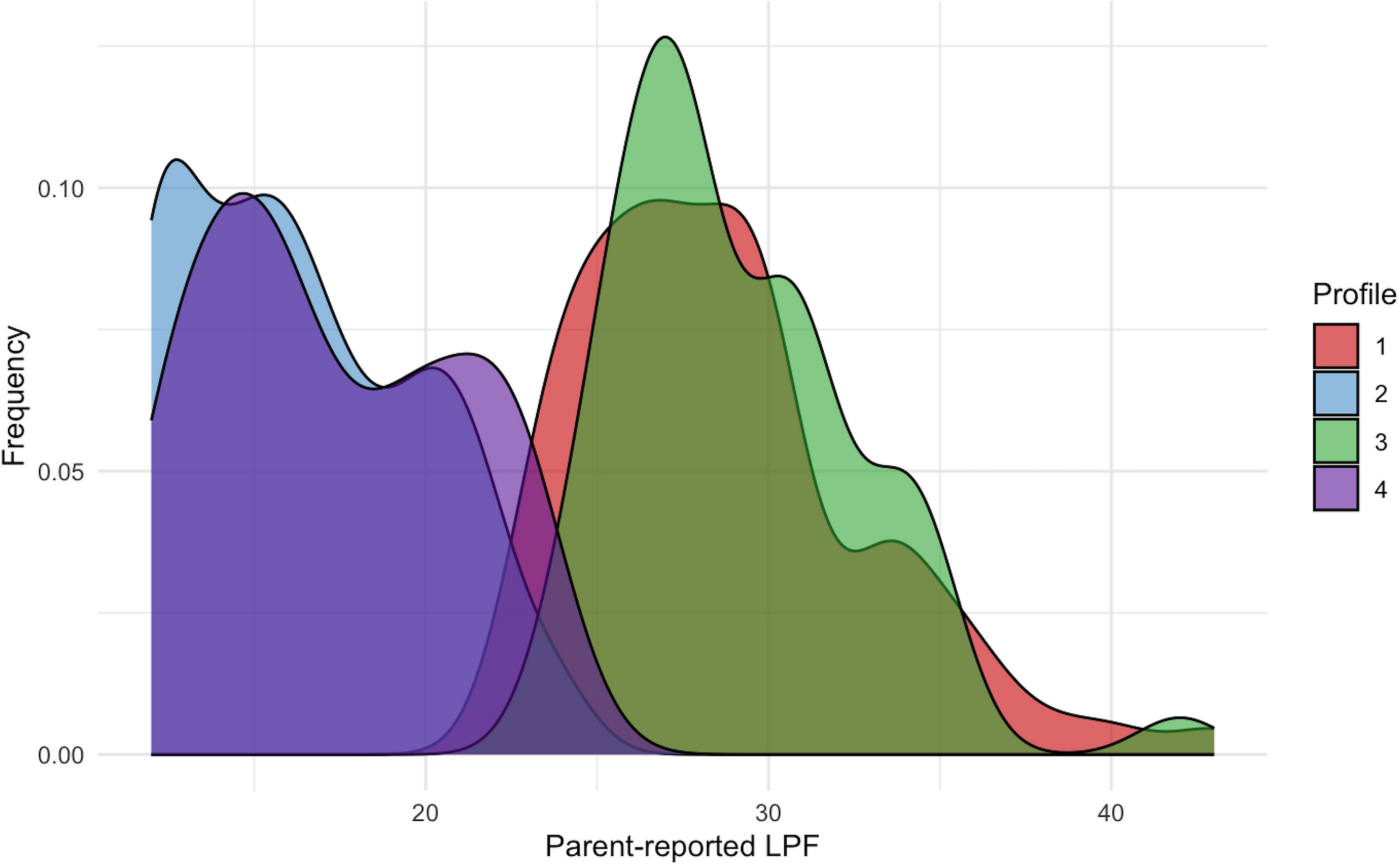
Density plots of parent-reported level of personality functioning within latent profiles. LPF, level of personality functioning.

Examination of the four-profile model (**Figures 1**, **2**) revealed (1): a “divergent/parent-high” profile with low child-reported impairment in LPF and high parent-reported impairment in LPF (2), a “convergent-low” profile with low child-reported impairment in LPF and low parent-reported impairment in LPF (3), a “convergent-high” profile with high child-reported impairment in LPF and high parent-reported impairment in LPF, and (4) a “divergent/child-high” profile with high child-reported impairment in LPF and low parent-reported impairment in LPF. Descriptive statistics for child-reported LPF, parent-reported LPF, child age, child sex, and parent sex separated by latent profile are presented in **Table 4**. Density plots of child-reported and parent-reported LPF within profiles are depicted in **Figure 3** and **Figure 4**, respectively. With regards to suggested cutoff scores on the LPFS-BF 2.0, in the divergent/parent-high profile, none of the child-reported scores were above the subclinical cutoff, while 75.0% of parent-reported scores were above the subclinical cutoff (and 23.68% were above the clinical cutoff). In the convergent-low profile, none of the child-reported or parent-reported LPF scores were above the subclinical cutoff. In the convergent-high profile, 100% of the child-reported LPF scores were above the subclinical cutoff (and 42.0% were above the clinical cutoff), and 88.0% of parent-reported LPF scores were above the subclinical cutoff (and 34.0% were above the clinical cutoff). In the divergent/child-high profile, 91.14% of the child-reported LPF scores were above the subclinical cutoff (and 30.38% were above the clinical cutoff), and none of the parent-reported LPF scores were above the subclinical cutoff.

**Table 4:** Within-profile descriptive statistics of level of personality functioning and demographics.

Profile	*N*	Child-reported LPF					Parent-reported LPF					Child age				Sex	
		*M*	*SD*	Min	Max	% above subclinical cutoff	*M*	*SD*	Min	Max	% above subclinical cutoff	*M*	*SD*	Min	Max	% female children	% female parents
1 Divergent/parent-high	76	19.557	3.451	12	25	0%	28.669	4.21	23	43	75.0%	11.684	1.257	10	14	64.47%	94.74%
2 Convergent-low	227	17.023	3.369	12	24	0%	16.393	3.448	12	24	0%	11.555	1.168	10	15	66.52%	89.87%
3 Convergent-high	50	30.868	3.783	26	42	100.0%	29.155	3.582	24	42	88.0%	12.38	1.176	10	14	83.67%	96.0%
4 Divergent/child-high	79	28.832	3.332	25	39	91.14%	17.308	3.664	12	24	0%	11.797	1.275	10	14	74.68%	89.87%

% above subclinical cutoff indicates percent of profile members with an LPF score greater than or equal to 26. LPF = level of personality functioning.

Pearson’s chi-square test indicated that there was a marginally significant difference in child sex across profiles (χ^2^ = 7.837, *df* = 3, *p* = .0495). *Post hoc* comparisons indicated that the convergent-high profile had a significantly greater proportion of female children compared to the divergent/parent-high profile (χ^2^ = 5.732, *p* = .017) and the convergent-low profile (χ^2^ = 5.926, *p* = .015), although these were not significant with a Bonferroni-corrected alpha level (ɑ = .008). Fisher’s exact test indicated that profiles did not differ significantly by parent sex (*p* = .388). ANOVA indicated that profiles differed significantly by child age, with Tukey’s HSD test indicating that children in the convergent-high profile were significantly older (*p_adj_* <.05) than children in all other profiles. Child age and sex were examined as covariates in further analyses.

Pearson’s chi-square test indicated that there was a significant association between study membership and profile membership (χ^2^ = 27.988, *p* <.001). We examined standardized residuals, applying Bonferroni correction for twelve tests, resulting in a corrected α of.004 and a critical value of 2.865. Examination of standardized residuals revealed only one residual with a magnitude greater than this critical value, which indicated that participants in the first study (pre- and early adolescent girls with and without attention-deficit/hyperactivity disorder) were disproportionately more likely to be in the convergent-high profile compared to participants in the other two studies. Given that older child age was significantly associated with membership in the convergent-high profile, we then examined the relationship between child age and study membership to determine whether child age might help account for this finding. Child age was significantly different across studies (*F*[2,429] = 16.89, *p* <.001). Children in the first study were significantly older than children in the second study (Tukey’s HSD = 0.773, *p* <.001) and children in the third study (Tukey’s HSD = 0.973, *p* <.001). Child age did not differ between the second and third study (Tukey’s HSD = -0.20, *p* = .298). Therefore, child age may help to account for the fact that participants in the first study were disproportionately more likely to be in the convergent-high profile, though other differences in study characteristics may have also contributed to this disproportionality.

### Associations of latent profiles with outcomes

Descriptive statistics of all outcome variables separated by latent profile are presented in **Table 5**. ANCOVA was conducted with profile membership, child age, and child sex predicting parent-reported caregiver strain. Results indicated that there was a significant difference in caregiver strain between profiles (*F* (5,425) = 49.884, *p* <.001, *R*^2^ = .370). Child age and sex were not significant predictors of caregiver strain (*p* = .053 and *p* = .853 respectively). Tukey’s HSD test (**Table 6**) indicated that the convergent-high and divergent/parent-high profiles did not differ significantly in caregiver strain, but were each associated with significantly greater caregiver strain compared to the convergent-low and divergent/child-high profiles. The convergent-low and divergent/child-high profiles did not differ significantly in caregiver strain.

**Table 5:** Within-profile descriptive statistics of outcome variables.

Profile	*N*	Child impairment				Caregiver strain				% suicidal ideation	% suicide plan	% suicide gesture	% suicide attempt	% NSSI thoughts	% NSSI engaged
		*M*	*SD*	Min	Max	*M*	*SD*	Min	Max						
1 Divergent/parent-high	76	2.677	1.245	0	5.75	2.215	0.765	1	4.5	23.68%	9.21%	2.63%	5.26%	14.47%	5.26%
2 Convergent- low	227	0.793	1.004	0	5.25	1.309	0.421	1	3.583	7.93%	0.88%	3.97%	0.44%	7.489%	5.73%
3 Convergent- high	50	2.923	1.226	0.25	6	2.295	0.75	1	4.167	48.0%	18.0%	14.0%	8.0%	36.0%	28.0%
4 Divergent/child-high	79	1.074	1.01	0	4	1.459	0.469	1	3.083	29.11%	3.80%	8.86%	1.27%	30.38%	20.25%

NSSI, non-suicidal self-injury.

**Table 6:** Post hoc pairwise comparisons of profile differences by outcome.

Contrast	Caregiver strain (HSD)	Child impairment (HSD)	Suicidal ideation (χ^2^)	Suicide plan (odds ratio)	Suicide gesture (odds ratio)	Suicide attempt (odds ratio)	NSSI thoughts (χ^2^)	NSSI engaged (odds ratio)
Convergent-low vs. Divergent/parent-high	**-0.906**	**-1.884**	**13.751**	**.088**	1.512	.079+	3.505	1.078
Convergent-high vs. Divergent/parent-high	0.080	0.247	**7.745**	2.119	5.858+	1.516	**7.439**	**6.883**
Divergent/child-high vs. Divergent/parent-high	**-0.757**	**-1.602**	0.515	0.386	3.522	0.226	5.213+	**4.475**
Convergent-high vs. Convergent-low	**0.986**	**2.131**	**50.864**	**24.137**	3.898+	**19.250**	**29.80**	**6.426**
Divergent/child-high vs. Convergent-low	0.150	0.282	**22.503**	4.394	2.337	2.873	**26.106**	**4.164**
Divergent/child-high vs. Convergent-high	**-0.836**	**-1.849**	4.716+	.182+	0.60	0.150	0.440	0.647

Bold indicates significance after correcting for multiple comparisons. + indicates *p* <.05 but not significant after correcting for multiple comparisons. HSD, Tukey’s honestly significant difference. NSSI, non-suicidal self-injury.

ANCOVA was then conducted with profile membership, child age, and child sex predicting parent-reported child functional impairment and indicated that there was a significant difference in child functional impairment between profiles (*F* (5,426) = 60.878, *p* <.001, *R*^2^ = .417). Child age and sex were also significantly associated with child functional impairment, such that younger child age (b = -0.087, *p* = .043) and male sex (b = 0.293, *p* = .010) were associated with greater functional impairment. Just as with caregiver strain, Tukey’s HSD test (**Table 6**) indicated that the convergent-high and divergent/parent-high profiles did not differ significantly in child functional impairment, but were each associated with significantly greater impairment than convergent-low and divergent/child-high profiles. The convergent-low and divergent/child-high profiles did not differ significantly in child functional impairment.

Chi-square and Fisher’s exact tests indicated that profiles differed significantly on all suicide- and NSSI-related variables. *Post hoc* comparisons with a Bonferroni-corrected alpha level (ɑ = .008) indicated that the convergent-high profile had a significantly greater proportion of suicidal ideation, thoughts of NSSI, and engagement in NSSI compared to the divergent/parent-high profile and the convergent-low profile. The convergent-high profile also had a significantly greater proportion of suicide plans and attempts compared to the convergent-low profile. The divergent/child-high profile was associated with significantly more engagement in NSSI compared to the divergent/parent-high profile. Both the divergent/child-high profile and divergent/parent-high profile were differentiated from the convergent-low profile for suicidal ideation.

Given that children were significantly older and more likely to be female in the convergent-high profile compared to other profiles, GLMs with a binomial distribution including child age and sex as covariates were conducted to examine whether profile differences in suicide- and NSSI-related outcomes remained after accounting for age and sex, with the convergent-high profile as the reference group. GLMs indicated that previously observed differences between the convergent-high profile and other profiles remained significant when accounting for age and sex for all outcomes. Older child age was significantly associated with suicide plans (b = 0.545, *p* = .012) and suicide attempts (b = 0.654, *p* = .038). See **Supplementary Material** (**Supplementary Table 4**) for results of GLMs for each outcome.

## Discussion

The current study examined patterns of convergence and divergence in parent- and child-reported LPF in a large sample of pre- and early adolescents and their parents, and associations of these patterns with potentially clinically important outcomes. At a basic correlational level, concordance between parent- and child report on LPF mirrored findings from studies covering a wide range of psychopathology, including borderline personality disorder features, which converge to suggest a concordance rate of approximately.25 –.30 (22, 23, 45, 46). In the latent profile analysis, the best fitting model included four profiles, including two convergent and two divergent profiles. Aligning with hypotheses, the profile reflecting convergent reports on high impairment in LPF was the most consistently associated with higher caregiver strain, child functional impairment, and suicide- and NSSI-related thoughts and behaviors compared to other profiles. Nearly half of participants assigned to this convergent profile reported a history of active suicidal ideation, and more than a quarter reported having engaged in NSSI.

Findings with divergent profiles were also consistent with our hypotheses. Regarding parent-reported outcomes, the convergent-high and divergent/parent-high profiles had similar associations with caregiver strain and child impairment and were both more strongly associated with these outcomes compared to the divergent/child-high profile. The pattern of results for child-reported outcomes was more complex, and overall suggested that the convergent-high profile showed the strongest associations with suicide- and NSSI-related thoughts and behaviors, followed by the divergent/child-high profile, although the divergent/parent-high profile was also informative about suicidal ideation and suicide plans. Despite there not being statistically significant differences between the convergent-high and divergent/child-high profiles on these outcomes, the proportions of suicide-related thoughts and behaviors were 1.5 to 6 times as high in the convergent-high profile as the divergent/child-high profile.

Through the lens of the operations triad model (31), these informant discrepancies may reflect meaningful differences in parents’ and children’s insight into the child’s impairment in personality functioning. We first consider the finding that the divergent/parent-high profile, but not the divergent/child-high profile, predicted parent-reported caregiver strain and functional impairment. A traditional view of informant discrepancy might discount the child’s report of higher impairment in LPF (in the divergent/child-high profile) as invalid because it was not associated with child functional impairment or caregiver strain when the parent did not also report higher impairment. However, from a diverging operations perspective, it may be that the discrepancy in the divergent/child-high profile reflects poorer caregiver attunement to the child’s impairment in personality functioning. Caregivers may be less attuned to their child’s difficulties for a number of reasons. For example, children in the divergent/child-high profile may have more internalized or less easily observable difficulties in personality functioning, which could reduce the extent to which the parent perceives their child’s difficulties to cause their child to have social, academic, or interpersonal difficulties or contribute to the parent’s objective or subjective strain. Some aspects of impairment in personality functioning - such as identity incoherence and lack of stability, or excessively low or fluctuating self-esteem - could be less noticeable to parents compared to other aspects of personality functioning, such as conflict in close relationships or difficulty tolerating others’ perspectives. Caregivers of children with higher self-reported impairment in LPF may also be less attuned to psychopathology in their children due to caregiver burnout or challenges associated with their own mental health. Another related possibility is that caregivers in the divergent/child-high profile could have lower mentalizing capacity and therefore reflect less on both the internal and external experiences of their child (47). Reduced caregiver mentalizing may diminish the caregiver’s awareness of challenges their child encounters, in turn reducing the level of experienced caregiver strain and perceived child functional impairment. Higher impairment in LPF has also been shown to be associated with challenges in the parent-child relationship (see 50 for a review). Such challenges exacerbate the likelihood that parents may not be in tune with the internal struggles of their children. Turning to the suicide- and NSSI-related outcomes, we see that the divergent/child-high profile was indeed uniquely informative compared to the divergent/parent-high profile, particularly with regards to engagement in NSSI and thoughts of NSSI. This finding supports the validity of the child report and suggests that we cannot discount a child’s report of higher impairment in LPF compared to their parent’s report.

If the divergent/child-high profile might reflect cases in which the child’s impairment in personality functioning is more internalized or less readily observable to parents, what might the divergent/parent-high profile represent? It may be that this profile reflects cases in which the parent has observed aspects of the child’s impairment in personality functioning into which the child lacks insight. Some aspects of personality functioning - such as difficulty estimating the impact of behavior on others and poor mutuality in relationships - may be more readily observed by others than by oneself, particularly if impairment in personality functioning also contributes to difficulties in self-reflection and mentalizing capacity. Again, although one could argue in favor of discounting parent reports in the divergent/parent-high profile as having an overreporting bias across the child’s personality impairment and functional difficulties and the parent’s strain, the finding that the divergent/parent-high profile was as good of a predictor of *child-reported* suicidal ideation and suicide plans as the divergent/child-high profile suggests that the parent report in this profile indeed has clinical significance above and beyond an overreporting bias. Finally, the convergent-high profile, which demonstrated the strongest and most consistent associations across outcomes, may reflect cases in which the child’s impairment in personality functioning is relatively more severe, more observable to others, and more observable across different contexts, such that both parents and children are more aware of and more likely to agree on the level of impairment. This suggestion of higher severity is supported by the finding that both child-reported and parent-reported LPF scores were above the suggested subclinical and clinical cutoff scores for more children in the convergent-high profile compared to all other profiles.

These interpretations do not rule out the possible contributions of what the operations triad model would call compensating operations (31), when informant discrepancies do not reflect meaningful differences and instead reflect invalid or unreliable measurement or methodological differences. One important limitation to note is that distributions of LPF scores were partially overlapping between profiles, such that there was a degree of uncertainty in the assignment of participants to latent profiles and some participants (particularly those with middling total LPF scores ranging from 23-26) might reasonably be assigned to a different profile. This limits the extent to which we can generalize the pattern of outcomes associated with a given profile to each participant assigned to that profile. However, when taken together, the results in the current study provide preliminary evidence of the value of informant discrepancies in LPF and suggest that both divergent profiles do offer unique information about outcomes in the current study, such that neither parent nor child report can be discounted on the basis of the other report being discrepant. It is also worth noting that both child-reported LPF and parent-reported LPF were associated with nearly all outcomes at the bivariate level, providing further support for the value of both child and parent reports.

### Strengths, limitations, and future directions

Strengths of the current study include, first, the fact that the current study is the first of our knowledge to evaluate the utility and provide some evidence for the validity of a parent-reported LPFS-BF 2.0. With an internal consistency value of ɑ = .875, we provide initial data in support of the LPFS-BF 2.0 parent report and call for more validity work to be conducted on this version of the measure. Additional strengths of the study include representation of a wide range in LPF in the sample and the use of latent profile analysis to examine informant discrepancies. These aspects of the study improved our ability to identify distinct convergent and divergent profiles with potentially clinically meaningful differences. Further, the focus on the pre- and early adolescent period provides a unique contribution to the literature, as research on personality pathology during this developmental period is still lacking compared to research in older age groups, and this period represents a critical window for early detection and intervention for personality disorder given that personality disorder often onsets in adolescence.

Limitations of the current study include analytical limitations, aspects of the sample composition that may limit generalizability, and limitations related to measures. Regarding analytical limitations, our profile solution yielded partially overlapping profiles, due in part to the use of only two indicators (child- and parent-reported LPF) in the LPA. While the degree of uncertainty in profile assignment was determined to be acceptable, and findings were similar when removing participants with more considerable uncertainty in profile assignment, it is important to acknowledge that uncertainty in profile assignment may have biased findings in subsequent analyses. Further, while findings suggested that child age may in part account for the fact that participants in the first study were disproportionately more likely to be in the convergent-high profile compared to participants in the other two studies, it is possible that other differences in study characteristics may have contributed to this disproportionality and that sampling artefacts may have contributed to some of the observed differences in our analyses. Finally, Fisher’s exact test, which was used to examine associations of latent profiles with infrequent outcomes (e.g. suicide plan, suicide gesture, suicide attempt, and NSSI engagement), does tend to be conservative and therefore may have resulted in a reduction in power to identify true statistically significant differences on these outcomes, particularly in combination with Bonferroni correction for multiple comparisons.

Regarding aspects of the sample composition that may limit generalizability, the sample was oversampled for females, with a portion of the sample specifically identified based on a diagnosis of ADHD. Additionally, we did not explicitly include a sample of youth diagnosed with personality disorder, which could have bolstered the generalizability of this study to adolescents with personality pathology. However, it has been well-documented that individuals with a personality disorder diagnosis typically receive several other psychiatric diagnoses first (88–90). Moreover, despite clear empirical research demonstrating the onset of personality disorder during adolescence, the translation of this evidence to clinical practice is limited and individuals often do not receive the diagnosis until adulthood (17). Thus, despite not having a personality disorder diagnosis as the inclusion criteria, the inclusion of youth with general mental health problems likely captures individuals with emerging personality disorder, which is substantiated by the proportion of youth- and parent-reports exceeding the suggested subclinical and clinical cutoff scores on the LPFS-BF 2.0. Another limitation is that the sample included mostly non-Hispanic White participants, lacking the racial and ethnic diversity to generalize results to Hispanic and non-White populations. Findings on racial and ethnic differences in personality pathology broadly and LPF specifically have been mixed (91–93) and require further exploration to parse out true differences from differences in accurate detection, service use, and other potentially confounding factors.

Finally, there are two measure-related limitations to note. First, the measure of LPF captured current personality functioning, while suicide- and NSSI-related outcomes on the SITBI captured lifetime endorsement, such that directionality cannot be inferred from associations between the latent profiles and these outcomes. It may in fact be an interesting future direction to consider the potentially bidirectional relationship between parent-child discrepancies in reports of LPF and child-reported suicide- and NSSI-related outcomes. Second, although results of the current study suggest the informative value of parent-reported LPF for clinically meaningful outcomes, we modified the typically self-reported LPFS-BF 2.0 (74) for use with parents, though this parent report has yet to be validated. More work is needed to develop parent, teacher, peer, and other informant reports of LPF beyond the few existing measures (i.e. the parent-reported LoPF-Q 6–18 PR; 60).

Future research on informant discrepancies in LPF could focus on further elaborating the nature of these discrepancies and their interpretation. For example, we have suggested that these discrepancies could reflect differences in the extent to which the parent or child could be expected to have insight into the child’s difficulties in personality functioning, which could be further investigated by comparing the specific aspects of impairment in personality functioning reported by participants in the two divergent profiles. Further, although LPF is conceptualized as a unidimensional construct (58), it could be valuable to include separate self and interpersonal functioning scores, LPF domain scores, or individual LPF items as indicators in LPA to gain more insight into the extent to which and ways in which parent and child reports of LPF diverge at these levels, and to potentially reveal more nuanced informant discrepancy profiles. Future work including other informants, such as teachers or peers, and laboratory-based measures could also be valuable. These informants could provide additional reports of LPF, as well as reports on other outcomes to minimize shared method variance with parent and child reports, thereby reducing the contribution of compensating operations. In addition, it could be beneficial to explore whether and how informant discrepancies in LPF vary across developmental period (i.e. pre-adolescence, early adolescence, mid-adolescence, late adolescence) and population (i.e. community, outpatient, inpatient), as this could inform the interpretation of informant discrepancies in LPF in clinical practice.

## Conclusion

Personality disorder often onsets in adolescence, making pre- and early adolescence a critical developmental period for early detection and intervention. Consistent with best standards assessment, the results of the current study support the value of multiple informants’ perspectives in the assessment of LPF among pre- and early adolescents. As calls are being made to expand assessment and treatment for personality disorder among adolescents in routine clinical practice (14, 16, 56, 94–96), it is also worth advocating for the integration of both child and parent perspectives on the child’s personality functioning into this assessment process. When parent and child reports converge on high LPF, this may reflect higher psychiatric severity and risk for self-harm. When parent and child reports on LPF diverge, instead of discounting a discrepant report, clinicians will likely benefit from considering factors that might drive the discrepancy and inform approaches to treatment. Noting discrepancies in reported LPF when providing assessment feedback may also offer valuable insight to adolescent patients and their parents and facilitate communication and mutual understanding between patients and parents. Such an approach is consistent with best practice in evidence-based therapeutic assessment (97) and with evidence-based approaches to treating personality pathology in youth, which highlight the importance of making explicit the discrepancies between parents’ and youths’ understanding of challenges and their solutions (98–100).

## Data Availability

De-identified data can be made available upon request to dbabinski@pennstatehealth.psu.edu.
