# Supplementary material for: Parent-child discrepancies in reports of pre- and early adolescent level of personality functioning
Source: Front Psychiatry. 2026 Mar 5;17:1773598. doi: 10.3389/fpsyt.2026.1773598 (PMC12999937; doi:10.3389/fpsyt.2026.1773598)
Supplement: Supplementary file 1 [file DataSheet1.pdf]

LPF Discrepancy Supplementary Materials

Table S1

Within-Profile Descriptive Statistics of Level of Personality Functioning and Demographics After Removing Participants with Low Profile Assignment Probability

		Child-reported LPF					Parent-reported LPF					Child age				Sex	
Profile	*N*	*M*	*SD*	Min	Max	% above subclinical cutoff	*M*	*SD*	Min	Max	% above subclinical cutoff	*M*	*SD*	Min	Max	% female children	% female parents
1 divergent parent-high	67	19.141	3.360	12	25	0%	29.341	4.022	24	43	85.075%	11.612	1.302	10	14	62.69%	95.52%
2 convergent low	227	17.023	3.369	12	24	0%	16.393	3.448	12	24	0%	11.555	1.168	10	15	66.52%	89.87%
3 convergent high	49	30.967	3.755	26	42	100.0%	29.219	3.59	24	42	87.755%	12.347	1.165	10	14	83.67%	95.92%
4 divergent child-high	77	28.893	3.353	25	39	90.91%	17.135	3.546	12	23	0%	11.792	1.281	10	14	74.03%	90.91%

Note. Participants were considered to have a low probability of profile assignment if their assignment probability was lower than 0.5. % above subclinical cutoff indicates percent of profile members with an LPF score greater than or equal to 26. LPF = level of personality functioning.

Table S2

Within-profile Descriptive Statistics of Outcome Variables After Removing Participants with Low Profile Assignment Probability

		Child impairment				Caregiver strain									
Profile	*N*	*M*	*SD*	Min	Max	*M*	*SD*	Min	Max	% suicidal ideation	% suicide plan	% suicide gesture	% suicide attempt	% NSSI thoughts	% NSSI engaged
1 Divergent/ parent-high	67	2.781	1.221	0	5.75	2.274	0.775	1	4.5	23.88%	8.96%	1.49%	4.48%	13.43%	5.97%
2 Convergent-low	227	0.793	1.004	0	5.25	1.309	0.421	1	3.583	7.93%	0.88%	3.97%	0.44%	7.49%	5.73%
3 Convergent-high	49	2.927	1.238	0.25	6	2.293	0.757	1	4.167	46.94%	16.33%	14.29%	8.16%	36.74%	28.57%
4 Divergent/ child-high	77	1.060	1.016	0	4	1.443	0.462	1	3.083	29.87%	3.90%	9.09%	1.30%	31.17%	20.78%

Note. Participants were considered to have a low probability of profile assignment if their assignment probability was lower than 0.5. NSSI = non-suicidal self-injury.

Table S3

Post hoc Pairwise Comparisons of Profile Differences by Outcome After Removing Participants with Low Profile Assignment Probability

Contrast	Caregiver strain (HSD)	Child impairment (HSD)	Suicidal ideation (χ^2)	Suicide plan (odds ratio)	Suicide gesture (odds ratio)	Suicide attempt (odds ratio)	NSSI thoughts (χ^2)	NSSI engaged (odds ratio)
Convergent-low vs. Divergent/parent-high	**-0.965**	**-1.989**	**13.156**	**0.090**	2.689	0.093+	2.445	0.955
Convergent-high vs. Divergent/parent-high	0.019	0.145	6.464+	1.940	10.637+	1.827	**8.098**	**6.275**
Divergent/child-high vs. Divergent/parent-high	**-0.831**	**-1.721**	0.567	0.408	6.433	0.274	5.929+	4.096+
Convergent-high vs. Convergent-low	**0.984**	**2.134**	**48.216**	**21.462**	3.990	**19.670**	**30.747**	**6.614**
Divergent/child-high vs. Convergent-low	0.134	0.267	**23.554**	4.512	2.403+	2.948	**27.263**	**4.302**
Divergent/child-high vs. Convergent-high	**-0.850**	**-1.867**	3.764	0.210+	0.603	0.150	0.417	0.650

Note. Participants were considered to have a low probability of profile assignment if their assignment probability was lower than 0.5. Bold indicates significance after correcting for multiple comparisons. + indicates $p < .05$ but not significant after correcting for multiple comparisons. HSD = Tukey's honestly significant difference. NSSI = non-suicidal self-injury.

Table S4

Estimates from Generalized Linear Models Examining Associations of Latent Profiles, Child Sex, and Child Age with Suicide- and Non-suicidal Self-injury-related Outcomes

Predictor	Outcome: Suicidal ideation	Suicide plan	Suicide gesture	Suicide attempt	NSSI thoughts	NSSI engaged
Divergent/parent-high (vs. Convergent-high)	**-0.981**	-0.559	**-1.833**	-0.342	**-1.073**	**-1.836**
Convergent-low (vs. Convergent-high)	**-2.258**	**-2.925**	**-1.442**	**-2.794**	**-1.821**	**-1.707**
Divergent/child-high (vs. Convergent-high)	-0.733	**-1.540**	-0.583	-1.801	-0.182	-0.317
Child age	0.136	**0.545**	-0.165	**0.654**	0.090	0.254
Child sex	-0.036	0.429	-0.468	1.032	-0.222	0.115

Note. Bold indicates $p < .05$. The convergent-high profile was used as the reference group for effects of other profiles. Generalized linear models were conducted using a binomial distribution. NSSI = non-suicidal self-injury.
